# A compressed sensing multi-slice cine CMR approach for the accurate assessment of left ventricular volumes and function

**DOI:** 10.1186/1532-429X-16-S1-P376

**Published:** 2014-01-16

**Authors:** Gabriella Vincenti, Davide Piccini, Pierre Monney, Jerome Chaptinel, Tobias Rutz, Simone Coppo, Michael O Zenge, Michaela Schmidt, Mariappan S Nadar, Pascal Chevre, Matthias Stuber, Juerg Schwitter

**Affiliations:** 1 Division of Cardiology and Cardiac MR Center, University Hospital of Lausanne (CHUV), Lausanne, Switzerland; 2 Advanced Clinical Imaging Technology, Siemens Healthcare IM BM PI, Lausanne, Switzerland; 3 Department of Radiology, University Hospital (CHUV) and University of Lausanne (UNIL)/Center for Biomedical Imaging (CIBM), Lausanne, Switzerland; 4 MR Applications and Workflow Development, Healthcare Sector, Siemens AG, Erlangen, Germany; 5 Siemens Corporate Technology, Princeton, New Jersey, USA; 6 Department of Radiology, University Hospital Lausanne (CHUV), Lausanne, Switzerland

## Background

CMR is generally accepted as the gold standard for left ventricular (LV) volumes and function assessment. The conventional CMR approach involves several breath-holds to cover the entire heart with short-axis acquisitions. Recently, compressed sensing (CS) techniques emerged as a means to considerably accelerate data acquisition. CS principally relies on: 1) transform sparsity, 2) incoherence of undersampling artifacts, and 3) nonlinear reconstruction. PURPOSE: To compare a novel CS-based single breath-hold multi-slice cine technique with the standard multi-breath-hold technique for the assessment of LV volumes and function.

## Methods

**Figure 1:**
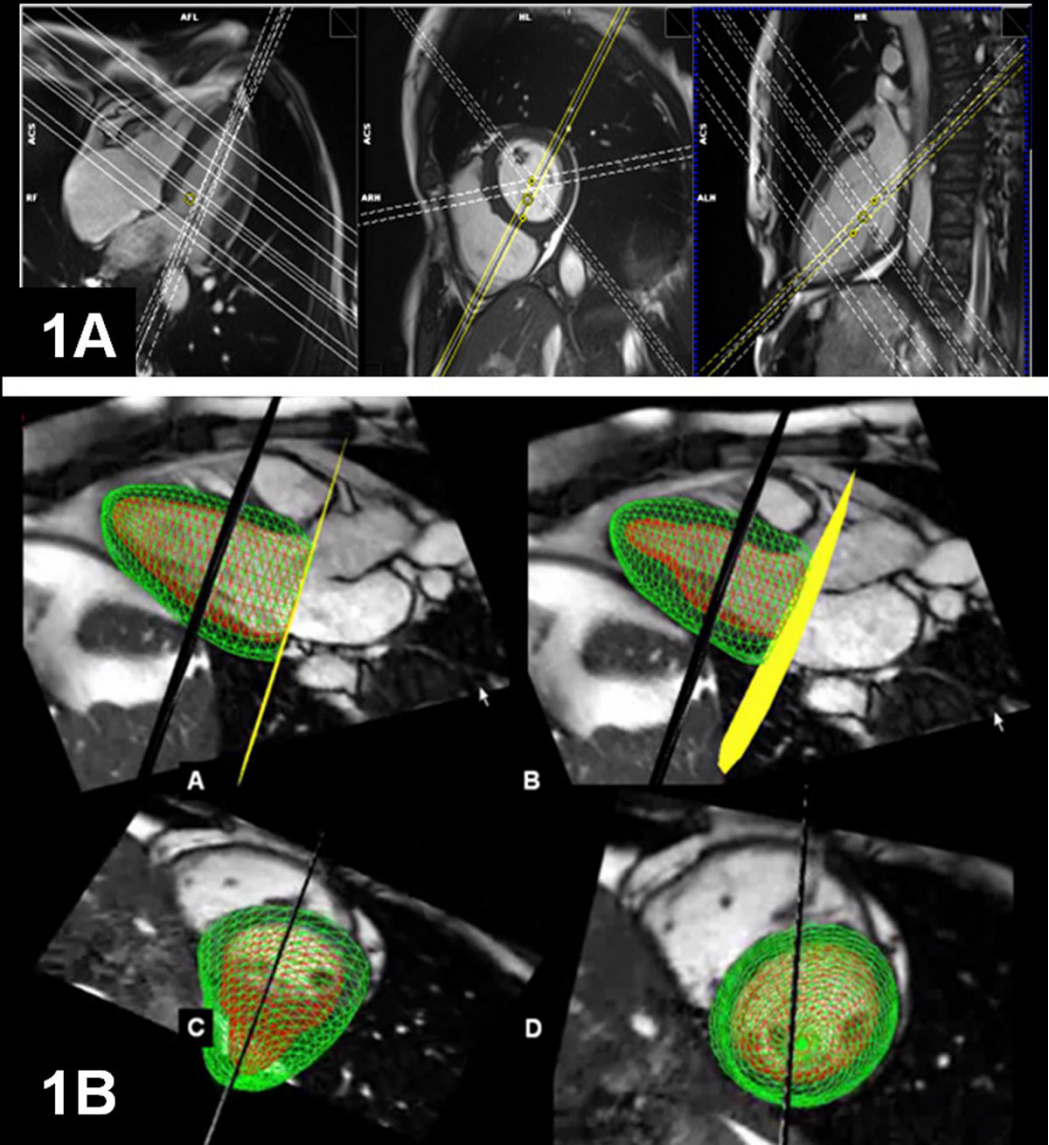


The novel single breathhold multi-slice CS cine sequence was implemented on a 1.5T MAGNETOM Aera (Siemens Healthcare, Germany). It realizes incoherent sampling by distributing the readouts pseudo-randomly on the Cartesian k space grid and by applying a pseudo-random offset from frame-to-frame during prospective ECG-triggering. Thus, 3 long-axis and 4 short-axis slices (Figure 1A) were acquired in a single breath-hold of 14 heart beats (temporal/spatial resolution: 30 ms/1.5 × 1.5 mm2, respectively, acceleration factor: 11.0). The CS cine data were analyzed by the Siemens Argus 4DVF software (Figure 1B) which is based on a 3D LV-model that takes the cyclic motion of the mitral valve plane (yellow plane in 2B) into account. For comparison, a conventional stack of cine SSFP images was acquired (temporal/spatial resolution 40 ms/1.2 × 1.6 mm2, slice thickness: 8 mm, gap: 2 mm) and analyzed by the Siemens Argus VF software. As a reference for the LV stroke volume (LVSV), the aortic flow (AoFlow) was measured by a phase-contrast acquisition (temporal/spatial resolution 40 ms/1.8 × 1.8 mm2).

## Results

**Figure 2:**
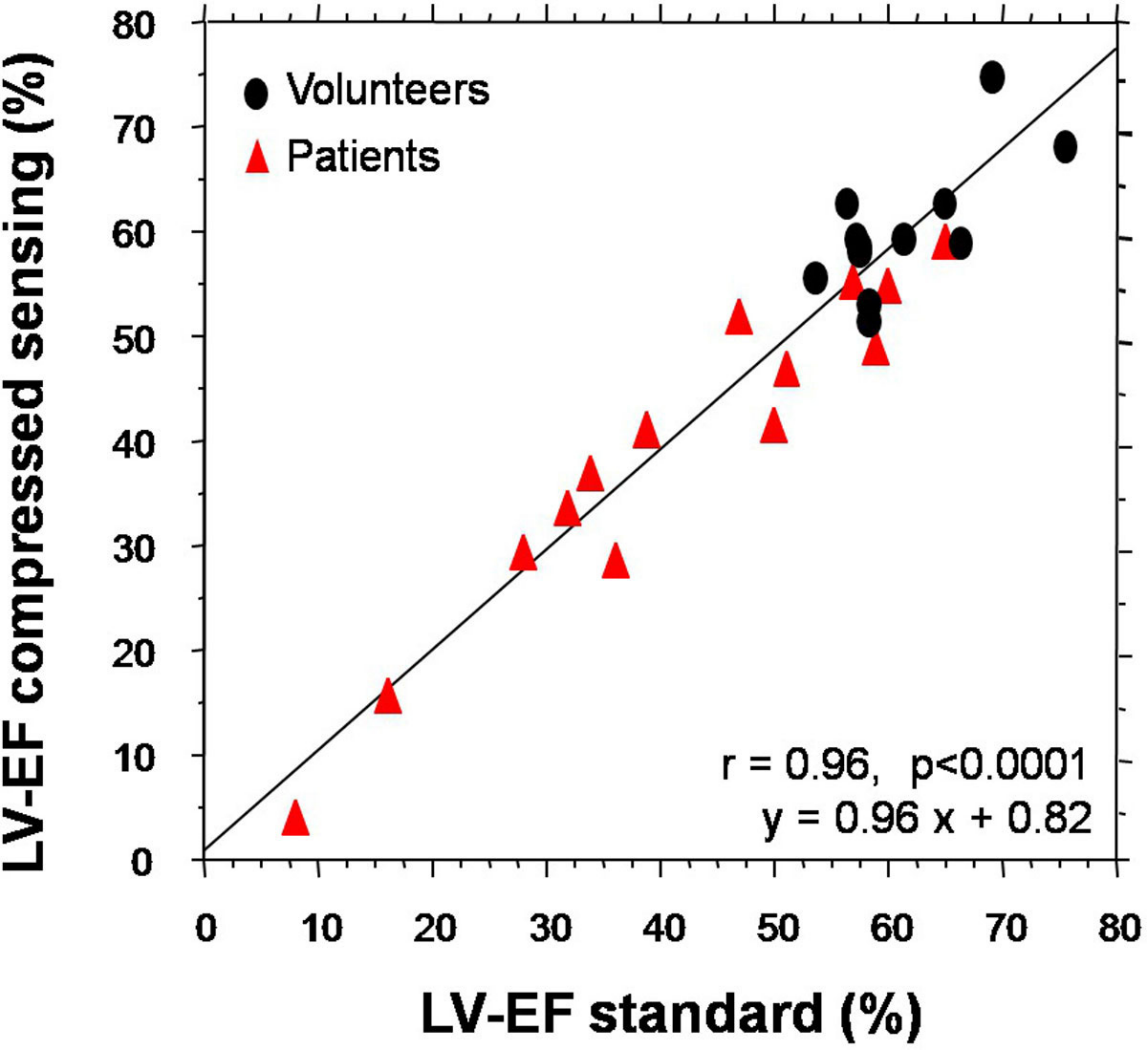


Twelve volunteers (75% male, age 33 ± 7 y) and 14 patients (85% male, age 67 ± 10 y) with different LV pathologies were studied. Good image quality of the single breathhold multi-slice CS acquisitions was obtained in 10/12 volunteers and 12/14 patients and all CS data were of adequate quality for 4D-analysis. LVEFCS and LVEFStandard were similar (49.3 ± 16.4% vs 50.6 ± 16.4%, respectively, p = 0.17) with an excellent correlation (Figure 2). Correlation of LVSVCS with AoFlow was also excellent (r = 0.93, p < 0.0001). For LVSVStandard the correlation was r = 0.70 (p < 0.01). Bland-Altman analyses showed underestimation of CS vs standard by 10.5 ml and 7.2 ml for LV end-diastolic (p < 0.005) and LV stroke volume (p < 0.002), respectively, no difference for LV end-systolic volume (p = ns).

## Conclusions

This is the first clinical experience with a novel CS-based cine sequence. These preliminary results demonstrate the potential of this fast single breath-hold technique to replace the multi-breath-hold standard technique for the assessment of LV volumes and function.

## Funding

D. Piccini, M.Zenge, M.Schmidt and M. Nadar are Siemens employees. Other funding than that was not received.

